# Pedicled Omental Onlay Flap for Post-Traumatic Intrahepatic Major Ductal Injury

**Published:** 2013-10-16

**Authors:** Simmi K Ratan, Anita Gangurde, Shandip K Sinha, Sudhir Singh, Barjesh Chander Sharma, Satish K Aggarwal

**Affiliations:** Department of Pediatric Surgery, Maulana Azad Medical College and Lok Nayak Hospital.; Department of Pediatric Surgery, Maulana Azad Medical College and Lok Nayak Hospital.; Department of Pediatric Surgery, Maulana Azad Medical College and Lok Nayak Hospital.; Department of Pediatric Surgery, Maulana Azad Medical College and Lok Nayak Hospital.; Department of Gastroenterology, GB Pant Hospital, New Delhi, India-110002.; Department of Pediatric Surgery, Maulana Azad Medical College and Lok Nayak Hospital.

**Keywords:** Post traumatic intra-hepatic bile duct, Omental flap, Bile leak

## Abstract

We report a 5-year-old girl who presented with post traumatic biliary leakage that failed to respond to conservative management for two weeks. Surgical exploration in the third week revealed a partially healed 5 cm long hepatic laceration in the right lobe of the liver. Bile was found leaking through a rent in the major right intra-hepatic duct at the apex of liver laceration. A pedicled onlay omental flap was used to buttress this rent as direct closure was not possible due to friable tissue. The child recovered uneventfully.

## INTRODUCTION

The overall incidence of biliary complications following blunt hepatic trauma for all grades of injury varies from 2.8% to 7.4%.[1] Mostly, such biliary leaks are from smaller hepatic ducts, are self-limiting, and often respond to conservative management. Other complications of pediatric blunt hepatic trauma include bilioma, hepatic artery pseudoaneurysm, and necrosis of gallbladder.[2] We report a girl in whom intrahepatic major bile duct injury following blunt abdominal trauma failed to heal with initial ultrasound guided peritoneal drainage and endoscopic intervention. She finally required surgical intervention.

## CASE REPORT

A 5-year-old hemodynamically stable girl with liver laceration following blunt abdominal trauma was initially managed conservatively. CT scan confirmed liver injury (Fig. 1). On tenth day of admission she developed gross abdominal distension, fever and ileus. Abdominal sonography (USG) confirmed presence of fluid in the abdominal cavity. Ultrasound guided pigtail catheter was inserted that drained 800 ml of bile. MRCP showed segment V liver injury with possibility of bile duct injury. ERCP revealed a leak from the right intrahepatic duct. Endoscopic stenting was tried but could not be done. Hence a papillotomy was performed. Despite this her fever continued and the collection became larger. An exploratory laparotomy was then performed, which revealed gross biliary peritonitis and a 5cm long laceration in the right lobe of liver. Bile was seen to be leaking from the apex of the laceration, from a 2 mm wide non-circumferential rent of right intrahepatic bile duct. The tissue on both sides of the laceration was quite friable, hence suture closure of the rent was considered inappropriate. A pedicled omentum flap was mobilized and packed into the laceration and tacked with few sutures. Peritoneal lavage was given, a tube drain placed in the hepatorenal pouch and the abdomen closed. Post operatively, the drain output gradually decreased and the child improved. The drain was removed on 6th postoperative day. Liver function tests reached normal levels over the next week. The child is doing well at a 2 year follow up (Fig. 2).

**Figure:**
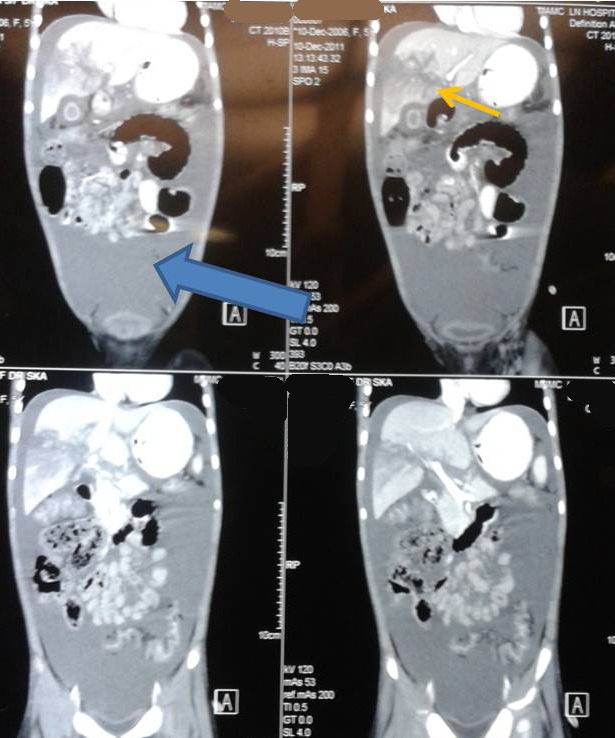
Figure 1:CT abdomen showing hypoechoic hepatic laceration (marked with thin arrow) with fluid collection (marked with thick arrow) in peritoneal cavity.

**Figure:**
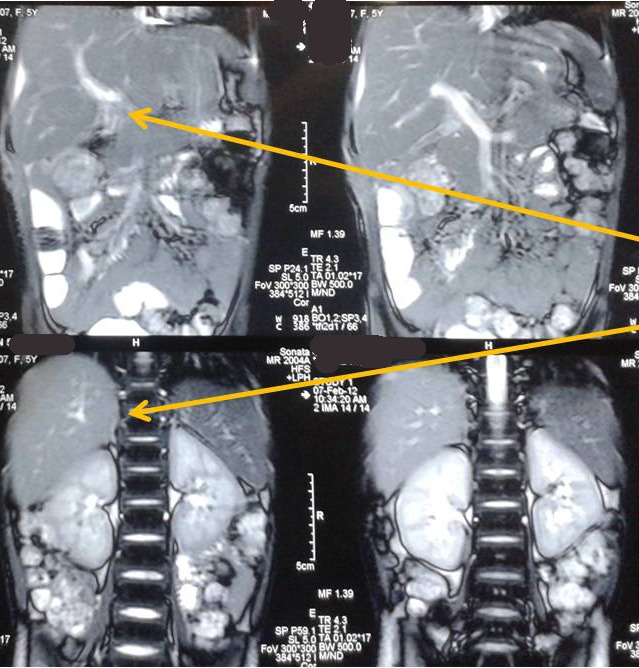
Figure 2:CT abdomen of the same patient at 6 months post injury, showing healed laceration (marked with arrow), with no intra-hepatic stasis/leak.

## DISCUSSION

In our patient, the bile leak became clinically obvious at the end of first week and was preceded by a period of ileus. Delayed presentation of such leaks is known and is due to a slow ischemic necrosis of the bile duct following blunt trauma. The diagnosis thus gets obvious on finding bile at peritoneal drainage though radio nucleotide studies can be employed for this purpose.[3-5]. In our patient a lot of necrosis in the hepatic parenchyma was found surrounding the leaking bile duct at exploration.

While managing bile ductal injuries in children, the emphasis is laid on the non-operative intervention which succeeds in most patients.[4,5] The non operative interventions include endoscopic papillotomy and/or stent placement with or without percutaneous interventions. However, Roech et al have expressed their concern about management of such leaks using conservative management alone.[6] They had to recourse to intrahepatic biliary reconstruction in one child and segmental resection in another with intrahepatic bile leak despite enough period of observation. Similar experience has also been stated by Moultan and Verran who had to subject the children with such injuries to variable extent of surgical exploration whenever non-operative treatment failed.[5,7] This ranged from minilaparotomy with peritoneal drainage to intrahepatic biliary reconstruction where hepatic tissue had good vascularity or even a segmental resection.[7]

In our patient fair period of observation and endoscopic intervention were employed but nothing beyond papillotomy could be done. Since the leak did not stop. Intrahepatic biliary reconstruction was not possible due to the surrounding devitalized hepatic parenchyma. At the same time, in view of poor general condition of the child even a segmental hepatic resection did not appear to be a safe option. Hence vascularized omental flap was considered as the duct was not completely transected. The approach has to be individualized.[5] In our patient pedicled omental onlay flap gave good result. Segmental resection is more often used among adults.[8]

Omentum is the ‘policeman of the abdominal cavity’ due to its high macrophage content. It has been extensively used to augment healing of the suture lines both in abdominal and extra-abdominal reconstructive surgeries. It has a rich blood supply. Omentum also helps in removing edema fluid and toxic substances due to its potent lymphatics.[9] Omental pedicled flaps have been described frequently to seal minor post traumatic/operative liver surface ooze and small biliary leaks. A recently published article in Chinese language describes the use of pedicled omentum for non-circumferential bile duct rent in two adult patients with Mirzzi syndrome.[10] The same was used in index case. Conservative management generally suffices in children with blunt hepatic injury resulting in bile leak. Omental pedicled flap can be offered in cases where such strategy fails and patient may not be in a condition to undergo major resection.

## Footnotes

**Source of Support:** Nil

**Conflict of Interest:** None declared

